# Caffeine blocks disruption of blood brain barrier in a rabbit model of Alzheimer's disease

**DOI:** 10.1186/1742-2094-5-12

**Published:** 2008-04-03

**Authors:** Xuesong Chen, Jeremy W Gawryluk, John F Wagener, Othman Ghribi, Jonathan D Geiger

**Affiliations:** 1 Department of Pharmacology, Physiology and Therapeutics, School of Medicine and Health Sciences, University of North Dakota, 501 N. Columbia Rd., Grand Forks, ND 58203, USA

## Abstract

High levels of serum cholesterol and disruptions of the blood brain barrier (BBB) have all been implicated as underlying mechanisms in the pathogenesis of Alzheimer's disease. Results from studies conducted in animals and humans suggest that caffeine might be protective against Alzheimer's disease but by poorly understood mechanisms. Using rabbits fed a cholesterol-enriched diet, we tested our hypothesis that chronic ingestion of caffeine protects against high cholesterol diet-induced disruptions of the BBB. New Zealand rabbits were fed a 2% cholesterol-enriched diet, and 3 mg caffeine was administered daily in drinking water for 12 weeks. Total cholesterol and caffeine concentrations from blood were measured. Olfactory bulbs (and for some studies hippocampus and cerebral cortex as well) were evaluated for BBB leakage, BBB tight junction protein expression levels, activation of astrocytes, and microglia density using histological, immunostaining and immunoblotting techniques. We found that caffeine blocked high cholesterol diet-induced increases in extravasation of IgG and fibrinogen, increases in leakage of Evan's blue dye, decreases in levels of the tight junction proteins occludin and ZO-1, increases in astrocytes activation and microglia density where IgG extravasation was present. Chronic ingestion of caffeine protects against high cholesterol diet-induced increases in disruptions of the BBB, and caffeine and drugs similar to caffeine might be useful in the treatment of Alzheimer's disease.

## Introduction

The blood-brain barrier (BBB), a physical and metabolic barrier between the central nervous system and the systemic circulation, helps regulate and protect the microenvironment of brain [1,2]. BBB breakdown occurs in a variety of neurological disorders including brain trauma [3], stroke [4], multiple sclerosis [5], HIV-1 dementia [6], Alzheimer's disease [7] and Parkinson's disease [8]. Changes in cholesterol homeostasis and high dietary cholesterol have been implicated in some of these neurological disorders (especially stroke and Alzheimer's disease) and have been shown to increase BBB leakage [9,10]. Because BBB functions to protect the central nervous system and disruption of BBB precedes neurological disorders such as stroke [11] and Alzheimer's disease [12], increased BBB leakage resulting from cholesterol-enriched diets might underly, at least in part, these neurological disorders.

Recent epidemiological and experimental studies indicate that caffeine, when administered chronically, has beneficial effects against a number of neurovascular disorders including stroke and Alzheimer's disease [13-20]. We [21,22] and others [18] have demonstrated beneficial effects of caffeine in early onset models of Alzheimer's disease. Here, we used rabbits fed a cholesterol-enriched diet as a model for sporadic Alzheimer's disease where problems with BBB integrity have been noted [9,10]. These studies were conducted mainly using olfactory bulbs, however confirmatory studies were conducted with hippocampus and cerebral cortex. Olfactory bulbs are a brain region with an intact BBB and are a region important for olfaction. In neurodegenerative diseases generally and Alzheimer's disease particularly, patients experience olfactory dysfunction [23]. Indeed, olfactory dysfunction has been suggested to be among the earliest symptoms of Alzheimer's disease and beta amyloid plaque and tau pathologies in the olfactory system have been reported in Alzheimer's disease [24]. Accordingly, olfactory bulbs are an important region with which to test the hypothesis that chronic ingestion of caffeine protects against high cholesterol diet-induced disruptions of the BBB.

## Materials and methods

### Animals

New Zealand white rabbits (1.5 to 2 years old) weighing 3 to 4 kg were used in the present study. Rabbits were randomly assigned to four groups; normal chow, normal chow + 3 mg/day caffeine, 2% cholesterol-enriched diet, 2% cholesterol-enriched diet + 3 mg/day caffeine. Caffeine was administered daily in 50 ml of drinking water starting from the beginning of these cholesterol feeding experiments. To help ensure that the rabbits drank the water in the absence or presence of caffeine, water was withheld for the prior 6 h and once the 50 ml were finished water was provided *ad libitum *for the remaining ~18 h/day. After 12 weeks of treatment, animals were anesthetized and perfused with PBS. Olfactory bulbs, hippocampi and cerebral cortices were dissected, frozen on a liquid nitrogen cooled surface, and stored at -80°C until taken for experimentation. All experiments were approved by the Committee for Animal Care and Use at the University of North Dakota.

### Evan's blue leakage assay

Evans blue dye (25 mg/kg) was injected i.p. and 3 h after injection plasma samples were collected via ear vein. Subsequently, rabbits were anesthetized heavily with ketamine/xylazine (50/5 mg/kg), and animals were perfused with 37°C oxygenated phosphate-buffered saline until colorless perfusion fluid was obtained from the right atrium. Following perfusion, brains were obtained quickly; olfactory bulbs, cerebral cortex and hippocampus were removed, weighed, and incubated for 72 h with formamide in the dark at room temperature. After incubation, samples were centrifuged at 10,000 × g for 10 minutes, supernatants were collected, and absorbance was measured at 620 nm. Evan's blue concentrations were calculated from standard curves. Final values were expressed as Evan's blue/specimen weight normalized to plasma Evan's blue concentration.

### Immunohistochemistry

Cryostat brain sections (14 μm) were fixed with acetone and stained for target proteins using antibodies to rabbit IgG (Jackson ImmunoResearch), fibrinogen (BD Pharmingen, clone 2C2-G7), CD31 (Abcam, clone JC/70A), vwf (Abcam, clone F8/86), and GFAP (Sigma, Clone G-A-5). Microglia were stained with biotin-conjugated Griffonia simplicifolia isolectin B4 (Molecular Probe). Fluorescent labeling with antibodies to ZO-1 (Zymed, clone ZO1-1A12) and occludin (Zymed, clone OC-3F10) were used for assessment of tight junction protein expression. For double immunostaining of IgG extravasation and the expression of occludin, ZO-1 or GFAP, sections were first incubated with biotin-conjugated goat anti-rabbit IgG and monoclonal antibodies to occludin, ZO-1 or GFAP, then incubated with Extravidin-TRITC (Sigma) and FITC-conjugated goat anti-mouse secondary antibodies. For double immunostaining of IgG extravasation and microglia, sections were first incubated with goat anti-rabbit IgG and biotin-conjugated Griffonia simplicifolia isolectin B4, then incubated with Texas Red-conjugated donkey anti-goat secondary antibody and Extravidin-FITC. Sections were examined by conventional (Leica) as well as confocal (Olympus) microscopy. Images were analyzed with Image J software.

### Immunoblotting

Olfactory bulb lysates containing equal amounts of protein were resolved with 10% SDS-PAGE and immunoblotted on membranes with antibodies to rabbit IgG, fibrinogen, GFAP, ZO-1, and occludin. Protein levels of rabbit IgG and fibrinogen in equal volumes of plasma were also resolved with SDS-PAGE and immunoblotted on membranes with antibodies to rabbit IgG and fibrinogen.

### Cholesterol measurements

Total serum cholesterol and high-density lipoproteins (HDL) were measured in venous blood collected from rabbit ear veins. Lipid levels were measured by standard techniques with an Olympus AU640 clinical analyzer.

### Caffeine measurements

Caffeine concentrations from blood were analyzed with high-performance liquid chromatography (HPLC). Proteins and lipids were removed with 2% trichloroacetic acid (TCA). After centrifugation at 12,500 × g for 15 min at 4°C, supernatants containing caffeine were collected. TCA in the supernatant was neutralized and separated with a mixture of tri-n-octylamine/dichloromethane (225:775). Samples were analyzed with a Supercosil C-18T column (4.6 × 150 mm, Supelco) using a mobile phase of 0.05 M KH_2_PO_4 _containing 4% acetonitrile at pH 3.8 run isocratically at 1.0 ml/min. Caffeine was detected with a photo-diode array 168 detector (Beckman Coulter) set to measure at 273 nm. Levels of caffeine were quantified by comparing peak heights and areas under the peaks with those obtained after sample spiking with caffeine and by comparison with values obtained with external caffeine standards.

### Statistical analyses

All data were expressed as means and SEM. Statistical significance for multiple comparisons was determined by two-way ANOVA and a Bonferroni post-hoc test. p < 0.05 was considered to be statistically significant.

## Results

### Caffeine blocks high cholesterol diet-induced increases in IgG and fibrinogen extravasation

**Figure 1:**
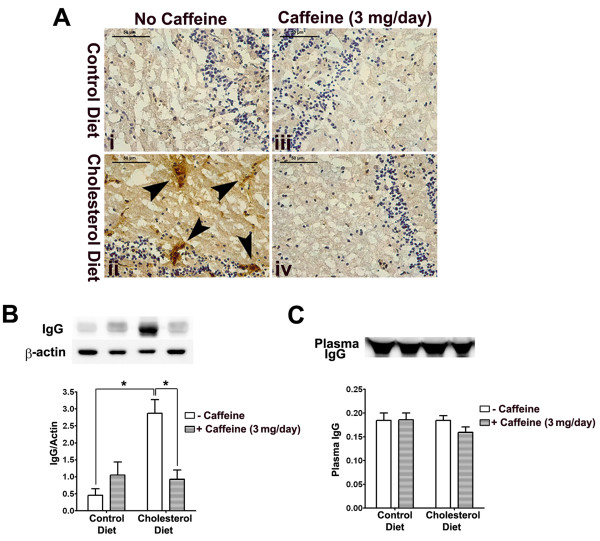
**Caffeine blocks high cholesterol diet-induced extravasation of IgG**. (A) Cholesterol-enriched diet increased extravasation of IgG in olfactory bulb and this effect was blocked by caffeine at the dose of 3 mg/day. Caffeine alone had no effect on extravasation of IgG in normal rabbit brain. Representative images taken from 2 rabbits in each group with 6 sections from each animal are shown. Bar = 50 μm. (B) Cholesterol-enriched diet significantly increased accumulation of IgG in olfactory bulb and this effect was blocked by caffeine at the dose of 3 mg/day. (C) Neither cholesterol-enriched diet nor caffeine changed significantly plasma levels of IgG. n = 4, *p < 0.05.

**Figure 2:**
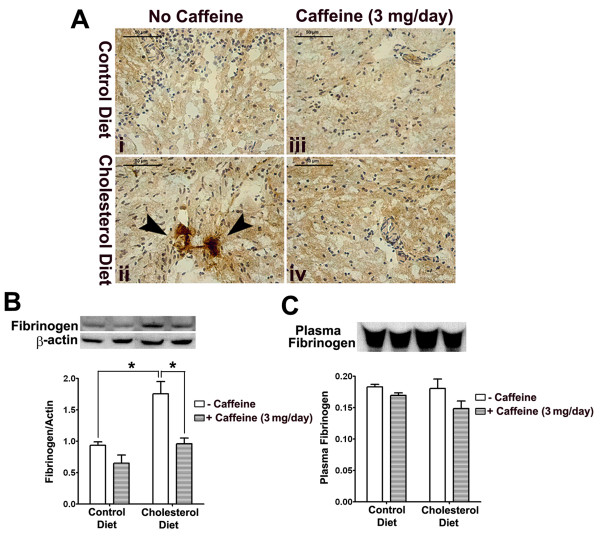
**Caffeine blocks high cholesterol diet-induced extravasation of fibrinogen**. (A) Cholesterol-enriched diet increased extravasation of fibrinogen in olfactory bulb and this effect was blocked by caffeine at the dose of 3 mg/day. Caffeine alone had no effect on extravasation of fibrinogen in normal rabbit brain. Representative images taken from 2 rabbits in each group with 6 sections from each animal are shown. Bar = 50 μm. (B) Cholesterol-enriched diet significantly increased accumulation of fibrinogen in olfactory bulb and this effect was blocked by caffeine at the dose of 3 mg/day. (C) Neither cholesterol-enriched diet nor caffeine changed significantly plasma levels of fibrinogen. n = 4, *p < 0.05.

Rabbits fed cholesterol-enriched diets have been used extensively to model cardiovascular disorders including atherosclerosis and more recently neurovascular disorders associated with sporadic Alzheimer's disease [25]. To investigate the effects of cholesterol-enriched diet and caffeine on BBB leakage, we first examined extravasation of endogenous IgG and fibrinogen. Because IgG and fibrinogen are not present normally in brain parenchyma, increases in the immunoreactivity of IgG and fibrinogen in brain indicate BBB leakage. Frozen sections of olfactory bulb were stained for IgG and fibrinogen and we found, in 2 of 6 rabbits examined from each group and in 6 sections from each animal, that the immunoreactivity of both IgG (Fig 1A) and fibrinogen (Fig 2A) was present perivascularly and that the cholesterol-enriched diet increased markedly IgG (Fig 1A) and fibrinogen (Fig 2A) extravasation. Increased extravasation of IgG, as examined by immunostaining, was present as well in cerebral cortex and hippocampus (data not shown). High cholesterol diet-induced increases in extravasation of IgG (Fig 1A) and fibrinogen (Fig 2A) in olfactory bulb were blocked by 3 mg of caffeine administered daily in drinking water for 12 weeks. In contrast, in olfactory bulb samples from control rabbits, caffeine alone had no effect on IgG (Fig 1A) and fibrinogen (Fig 2A) extravasation.

To confirm and extend the qualitative immunohistochemical results, we quantitatively evaluated the integrity of the BBB by measuring protein levels of IgG and fibrinogen using immunoblotting techniques. Consistent with our immunohistochemical results, the high cholesterol diet increased significantly (p < 0.05) protein levels of IgG (Fig 1B) and fibrinogen (Fig 2B) in olfactory bulb, and these increases were blocked completely and significantly (p < 0.05) by caffeine at the dose of 3 mg/day for 12 weeks. In control rabbits, caffeine did not change significantly the protein levels of IgG (Fig 1B) and fibrinogen (Fig 2B). To determine whether accumulation of IgG and fibrinogen in brain was due to increased levels of plasma IgG and fibrinogen possibly resulting from peripheral inflammation, we examined plasma levels of IgG and fibrinogen with immunoblotting and found that neither cholesterol-enriched diet nor caffeine changed significantly plasma levels of IgG (Fig 1C) and fibrinogen (Fig 2C).

### Caffeine blocks high cholesterol diet-induced leakage of Evan's blue dye

**Figure 3:**
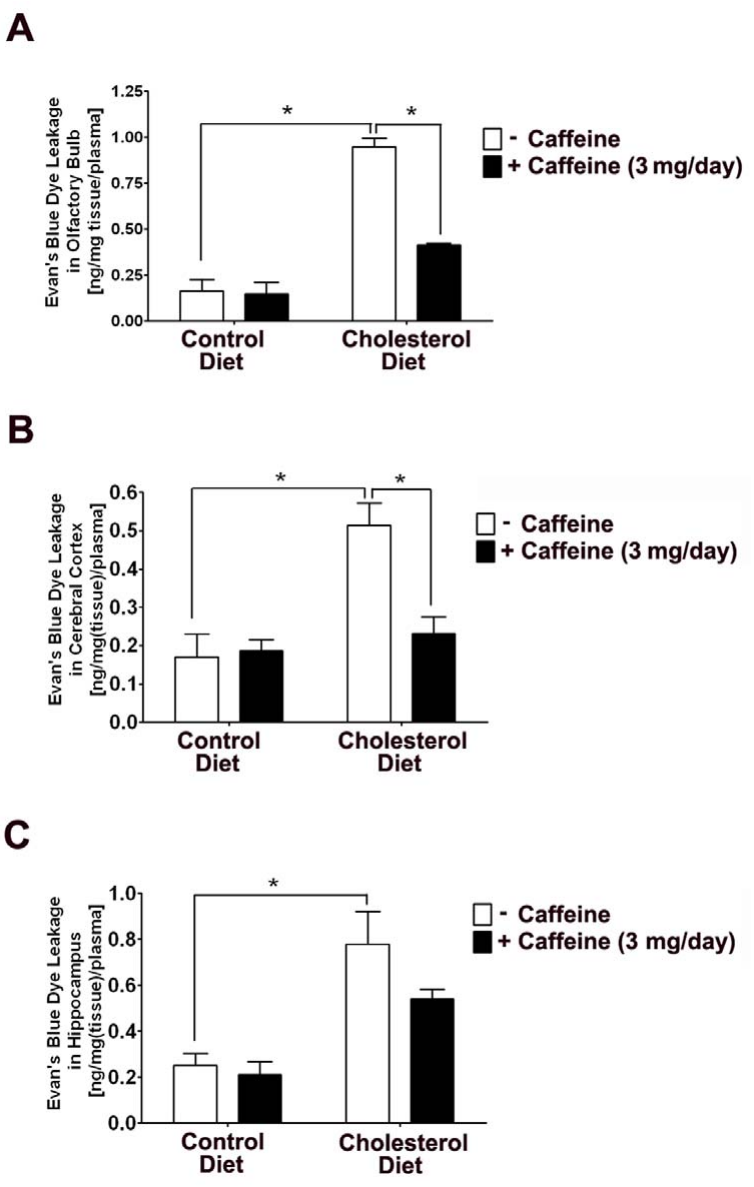
**Caffeine blocks high cholesterol diet-induced leakage of Evan's blue dye**. Cholesterol-enriched diet significantly increased leakage of Evan's blue dye in olfactory bulb (A), cerebral cortex (B) and hippocampus (C), and these effects were blocked by caffeine at the dose of 3 mg/day. n = 2, *p < 0.05.

We next evaluated BBB leakage using a quantitative Evan's blue dye leakage assay in 2 rabbits from each group and found that high cholesterol diet significantly increased leakage of Evan's blue dye in olfactory bulb (Fig 3A), cerebral cortex (Fig 3B) and hippocampus (Fig 3C). Ingestion of caffeine at the dose of 3 mg/day for 12 weeks attenuated high cholesterol diet-induced leakage of Evan's blue dye in olfactory bulb (Fig 3A), cerebral cortex (Fig 3B), and hippocampus (Fig 3C).

### Caffeine blocks high cholesterol diet-induced down-regulation of tight junction proteins

**Figure 4:**
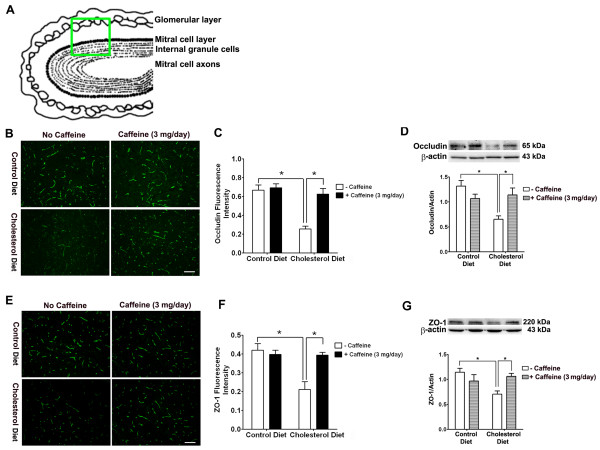
**Caffeine blocks high cholesterol diet-induced down-regulation of tight junction proteins**. (A) Schematic draft of a section of olfactory bulb, the green square indicated where the fluorescent images were taken for measures of the expression of occludin and ZO-1. (B) Decreased occludin immunostaining was observed in olfactory bulb from cholesterol-fed rabbits and this effect was blocked by caffeine. Caffeine alone had no effect on occludin immunostaining in normal rabbit brain. Representative images taken from 2 rabbits in each group with 6 sections from each animal are shown. Bar = 100 μm. (C) Quantitative data from B shows that high cholesterol diet significantly decreased occludin immunopositive staining in olfactory bulb, and this effect is blocked by caffeine. (D) Cholesterol-enriched diet decreased significantly protein levels of occludin, and these effects were blocked by caffeine at the dose of 3 mg/day. Caffeine alone did not significantly change protein levels of occludin in normal rabbit olfactory bulb (n = 4, *p < 0.05). (E) Decreased ZO-1 immunostaining was observed in olfactory bulb from cholesterol-fed rabbits and this effect was blocked by caffeine. Caffeine alone had no effect on ZO-1 immunostaining in normal rabbit brain. Representative images taken from 2 rabbits in each group with 6 sections from each animal are shown. Bar = 100 μm. (F) Quantitative data from E shows that high cholesterol diet significantly decreased ZO-1 immunopositive staining in olfactory bulb, and this effect is blocked by caffeine. (G) Cholesterol-enriched diet decreased significantly protein levels of ZO-1, and these effects were blocked by caffeine at the dose of 3 mg/day. Caffeine alone did not significantly change protein levels of ZO-1 in normal rabbit olfactory bulb. n = 4, *p < 0.05.

**Figure 5:**
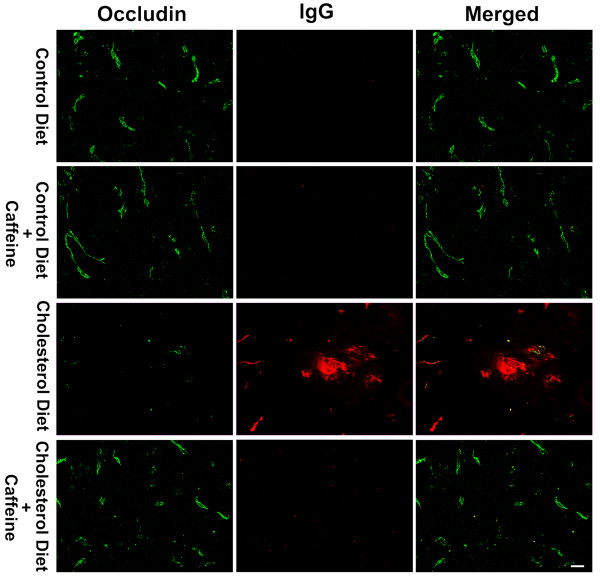
**Double immunostaining of IgG extravasation and the expression of occludin**. Cholesterol-enriched diet markedly decreased the immunopositive staining of occludin (green) at the sites where IgG extravasation (red) was present. These effects were blocked by chronic ingestion of caffeine. Representative images taken from 2 rabbits in each group with 6 sections from each animal are shown. Bar = 20 μm.

**Figure 6:**
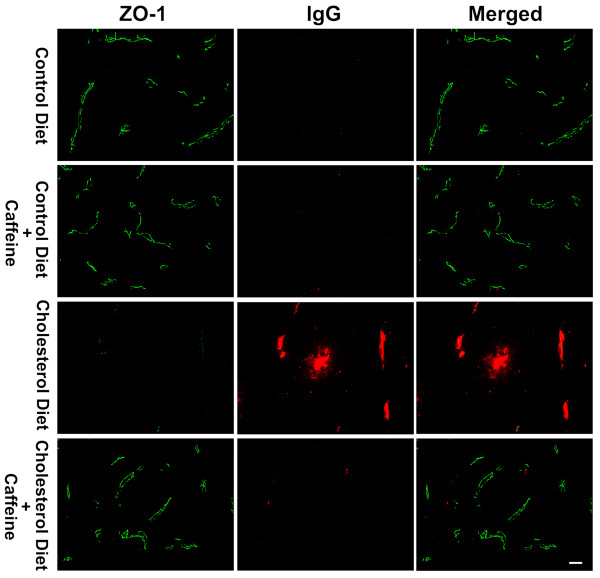
**Double immunostaining of IgG extravasation and the expression of ZO-1**. Cholesterol-enriched diet markedly decreased the immunopositive staining of ZO-1 (green) at the sites where IgG extravasation (red) was present. These effects were blocked by chronic ingestion of caffeine. Representative images taken from 2 rabbits in each group with 6 sections from each animal are shown. Bar = 20 μm.

The restrictive nature of the BBB is due mainly to tight junctions between adjacent endothelial cells. Stabilization of tight junctions involves a complex network of tight junction proteins such as occludin, claudins and zonula occludens (ZO) that link transmembrane proteins to the actin cytoskeleton [26]. Accordingly, we next determined the effects of cholesterol-enriched diet in the absence and presence of caffeine on expression levels of these proteins. Immunohistochemically, we observed, in 2 of 6 rabbits examined from each group and in 6 sections from each animal, significantly decreased occludin and ZO-1 immunostaining in olfactory bulb of cholesterol-fed rabbits, and this effect of cholesterol on these tight junction proteins was blocked by caffeine at the dose of 3 mg/day (Fig 4B, C, E and 4F). Protein levels of occludin and ZO-1, as determined by immunoblotting, were examined in control and cholesterol-fed animals. Caffeine blocked high cholesterol diet-induced decreases in the expression of occludin (Fig 4D) and ZO-1 (Fig 4G). Similar results were observed in hippocampus of cholesterol-fed and caffeine-treated cholesterol-fed rabbits (data not shown). We also examined the expression of tight junction proteins at the sites where BBB leakage was apparent using double fluorescent immunostaining. We observed that cholesterol-enriched diet decreased markedly the density of immunoreactive staining for occludin (Fig 5) and ZO-1 (Fig 6) at the sites where extravasation of IgG was present. These effects were all blocked by chronic ingestion of caffeine at the dose of 3 mg/day for 12 weeks.

### Caffeine does not affect plasma levels of cholesterol

In 4 rabbits from each group, we measured caffeine (μM) levels in serum using HPLC. Caffeine levels in rabbits fed control diet or cholesterol-enriched diet were not detectable. Caffeine levels were 5.3 ± 1.6 in the serum of rabbits fed control diet plus 3 mg/day caffeine, and were 6.3 ± 0.8 in rabbits fed 2% cholesterol-enriched diet plus 3 mg/day caffeine; these differences were not statistically significant. To investigate potential mechanisms by which caffeine protects against high cholesterol diet-induced disruption of BBB, we examined whether caffeine affects plasma cholesterol levels. We found that rabbits fed a cholesterol-enriched diet for 12 weeks exhibited a 10-fold increase in total plasma cholesterol concentration from 64 ± 7 mg/dl in controls (n = 6) to 644 ± 106 mg/dl in cholesterol-fed rabbits (n = 6). Caffeine did not affect significantly plasma cholesterol levels in rabbits fed control diet or cholesterol-enriched diet; values were 87 ± 12 mg/dl for control diet plus 3 mg/day caffeine animals and 593 ± 278 for cholesterol-enriched diet plus 3 mg/day caffeine animals. Plasma levels of high-density lipoproteins (HDL) decreased from 34 ± 4 mg/dl in controls to 14 ± 2 mg/dl in cholesterol-fed rabbits, and these levels were not affected significantly by caffeine; values were 40 ± 6 mg/dl in rabbits fed control diet plus 3 mg caffeine and 11 ± 3 mg/dl in rabbits fed cholesterol-enriched diet plus 3 mg caffeine.

### Caffeine blocks high cholesterol diet-induced increases in astrocyte activation and microglia density at the sites of BBB leakage

We examined the extent to which high cholesterol diet-induced increases in BBB leakage was associated with angiogenesis. Accordingly, sections of olfactory bulb were stained for the endothelial markers, protein platelet endothelial cell adhesion molecule-1 (CD31) and von Willebrand factor; no vascularization changes were noted in rabbits fed cholesterol-enriched diet and caffeine did not affect vascularization patterns in control or cholesterol-enriched diet fed animals (data not shown).

**Figure 7:**
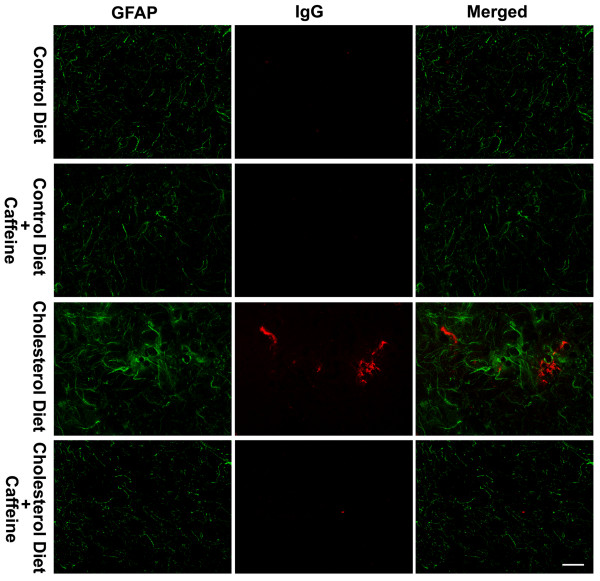
**Double immunostaining of IgG extravasation and the expression of GFAP**. Cholesterol-enriched diet markedly enhanced the immunopositive staining of GFAP (green) around the sites where IgG extravasation (red) was present. These effects were blocked by caffeine at the dose of 3 mg/day. Representative images taken from 2 rabbits in each group with 6 sections from each animal are shown. Bar = 20 μm.

**Figure 8:**
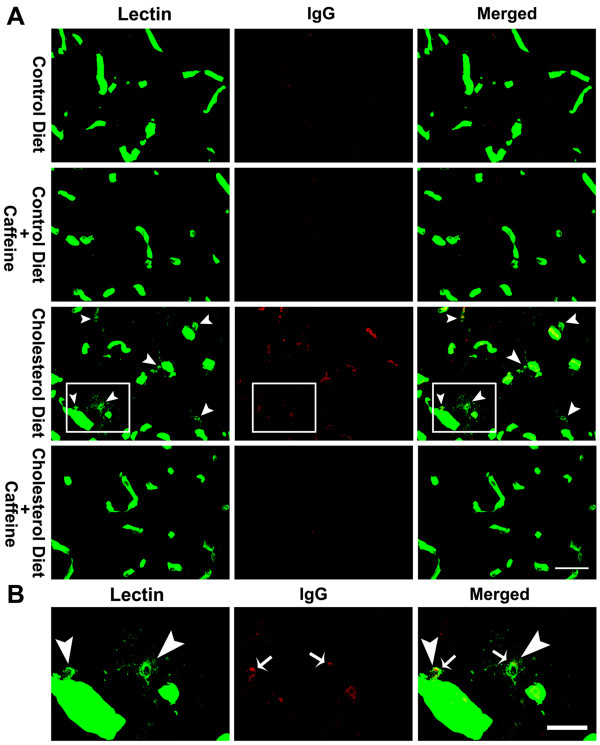
**Double staining of IgG extravasation and microglia**. (A) In addition to microglia, lectin staining also reveals blood vessels. Cholesterol-enriched diet markedly increased the numbers of microglia (green, arrow heads) and IgG extravasation (red). These effects were blocked by caffeine at the dose of 3 mg/day for 12 weeks. Representative images taken from 2 rabbits in each group with 6 sections from each animal are shown. Bar = 50 μm. (B) In a magnified view of the white boxes as shown in panel A, increased microglia (arrow heads) are co-distributed with perivascular immunopositive IgG staining (arrows) in olfactory bulb from cholesterol-fed rabbits. Bar = 20 μm.

BBB disruption and high dietary cholesterol have been shown to induce activate astrocytes and microglia [27-29]. Thus, we examined the involvement of astrocytes and microglia in our cholesterol-fed rabbit model. Activation of astrocytes was evaluated by immunostaining and immunoblotting for expression levels of the astrocyte marker glial fibrillary acidic protein (GFAP). We did not observe any overall changes in GFAP immunostaining and GFAP protein levels in olfactory bulb from rabbits fed cholesterol-enriched diet in the presence or absence of caffeine (data not shown). However, we did observe, in 2 of 6 rabbits examined from each group and in 6 sections from each animal, reactive astrocytes at the sites where extravasation of IgG was present (Fig 7), and these were not detected in rabbits fed control diet or in caffeine-treated animals. Using lectin staining as a measure of microglial density [27,30], we found, in 2 of 6 rabbits examined from each group and in 6 sections from each animal, that the cholesterol-enriched diet increased markedly the numbers of micrglia, and this effect was blocked by caffeine at the dose of 3 mg/day for 12 weeks (Fig 8). We also demonstrated that increased microglia in olfactory bulb from cholesterol-fed rabbits co-distributed with immunopositive staining of IgG perivascularly (Fig 8).

## Discussion

Here, we report that chronic ingestion of caffeine protects against disruption of the BBB, an early event in both acute and chronic neurodegenerative diseases [11,12]. We found that caffeine blocked high cholesterol diet-induced increased BBB leakage, decreased expression of endothelial cell tight junction proteins, increased activation of astrocytes, and increased microglia density in rabbit brain.

The BBB limits the entry of blood-borne pathogens, substances, drugs, and cells into brain parenchyma, and once disrupted can compromise synaptic and neuronal function [2,31,32]. One of the common features of neurological disorders such as stroke and Alzheimer's disease is BBB breakdown, and BBB disruption has been shown to precede neuronal damage in stroke [11] and Alzheimer's disease [12]. Thus, BBB breakdown might underlie these neurological disorders. Elevated levels of cholesterol have emerged as a risk factor for both stroke [33] and Alzheimer's disease [34] and others and we have shown that cholesterol-enriched diets induce pathological features of Alzheimer's disease such as learning deficit, increased Aβ plaque formation, hyperphosphorylation of tau, and neuronal cell death [10,25,35-37]. Cholesterol-enriched diet has also been shown to disrupt BBB [9,10]. Thus, cholesterol-enriched diets might contribute to the pathogenesis of Alzheimer's disease by virtue of its ability to compromise BBB integrity. Consistent with the above findings, the present study demonstrated that a cholesterol-enriched diet increased the leakage of the BBB as well as disrupted the integrity of the BBB in part by decreasing the expression of tight junction proteins.

Neurological disorders, such as stroke and Alzheimer's disease, take a huge toll on the elderly, but therapeutic interventions for these disorders are limited. Data from recent studies indicate that caffeine, the most commonly ingested psychoactive drug in the world, is protective against a number of acute and chronic neurological disorders including stroke and Alzheimer's disease [13-20]. However, little is known about the mechanisms whereby caffeine exerts its neuroprotective effects. Here, we showed, for the first time, that chronic ingestion of caffeine protects against disruption of BBB. The dose of caffeine (3 mg/day) used in this study for 3 to 4 kg rabbits is equivalent to an adult human weighing 70–80 kg consuming a single cup of coffee and is far less than the average caffeine consumption in the USA and Canada that is about 200 mg per person per day [38]. Furthermore, although the data were not included here we have evidence that a ten-fold higher dose of caffeine (30 mg/day) produces results virtually identical to the findings reported here. Both endogenous (IgG and fibrinogen) and exogenous (Evan's blue dye) markers were used to evaluate the effects of cholesterol-enriched diet in the absence and presence of caffeine on BBB leakage, and we found that caffeine blocked increased BBB leakage caused by the cholesterol-enriched diet. Furthermore, we demonstrated that caffeine blocked high cholesterol diet-induced down-regulation of the tight junction proteins, occludin and ZO-1, especially where BBB leakage were apparent. Our findings suggest that caffeine protects against BBB breakdown by keeping the expression levels of tight junction proteins from decreasing in this model. Since BBB disruption can compromise synaptic and neuronal function, our observation that caffeine protects against BBB disruption is consistent with the findings that caffeine intake protects against memory loss in aging and in Alzheimer's disease [19,20].

Similar to most other brain regions, the BBB in olfactory bulbs is normally intact and we could find no literature suggesting that the BBB in olfactory bulbs is 'leaky' under normal conditions. Based on our observations from our IgG and fibrinogen extravasation study, the BBB is indeed intact in olfactory bulbs from rabbits fed a control diet in the absence or presence of caffeine. Moreover, results from our Evan's blue dye leakage assay showed that rabbits fed control diet in the absence or presence of caffeine have similarly restrictive blood brain barriers in olfactory bulb, cerebral cortex and hippocampus. Our observation that caffeine blocks cholesterol-enriched diet induced Evan's blue dye leakage not only in olfactory bulb but also in cerebral cortex and hippocampus suggests that the effects of caffeine and cholesterol-enriched diet on BBB are general and not brain region specific.

Olfactory bulb was selected for these studies in part because olfactory dysfunction is one of the earliest symptoms experienced by patients living with Alzheimer's disease and olfaction tests have been investigated as an early diagnostic test [23,39-44]. Dysfunctions in olfaction are not limited to perceptual impairments and changes in olfactory thresholds, and beta amyloid plaque and tau pathology in the olfactory system have been reported in Alzheimer's disease [24]. Indeed, olfactory regions of brain including olfactory bulbs have some of the highest levels of neurofibrillary tangles and amyloid plaques in Alzheimer's disease [45,46]. It is relevant and potentially important that BBB dysfunctions were noted in olfactory bulbs (as well as hippocampus and cerebral cortex) in our model of Alzheimer's disease and that caffeine protected against these deleterious effects. We are not aware of any studies examining possible effects of caffeine on olfaction perception or thresholds.

Our observations that caffeine had no effects on plasma levels of total cholesterol and HDL indicate that caffeine protects against high cholesterol diet-induced disruption of BBB downstream of cholesterol. Angiogenesis and inflammation are major factors that could lead to BBB disruption [47,48]. The observations that neither cholesterol-enriched diet nor caffeine affect brain vascularization indicates that angiogenesis is not likely to play a major role in regulating BBB integrity in our animal model. BBB disruption induces astrocytes and microglia activation [29]. Therefore we examined the involvement of astrocyte and microglia in our animal model. We observed, in olfactory bulb from cholesterol-fed rabbits, site-specific astrogliosis where BBB leakage was apparent. In contract, astrogliosis was not observed in rabbits fed control diet or in caffeine-treated animals. We also found that the cholesterol-enriched diet increased the numbers of microglia, that the increased presence of microglia co-distributed with perivascular IgG, and that caffeine blocked these effects. These observations are consistent with previous reports that high dietary cholesterol induces activation of astrocyte and microglia [27,28,30]. Although the sequence of these events was not explored in the present study, our observation that activation of astrocytes and increase in the density of microglia occurred at sites where BBB leakage was apparent prompts us to speculate that cholesterol-enriched diet disrupts the BBB first and that the subsequent activation of astrocytes and increases in microglia density might be part of a potentially protective response. Most importantly, our studies demonstrated that caffeine is protective against high cholesterol diet-induced increases in BBB disruption, increases in astrocytes activation, and increases in microglia density. The protective effects of caffeine against high cholesterol diet-induced increases in BBB disruption might happen at the BBB *per se*, and the protective effects of caffeine against high cholesterol diet-induced increases in the density of astrocytes and microglia could be an indirect consequence of its protective effects against BBB disruption. On the other hand, it has been shown that caffeine (and adenosine) can regulate neuroinflammation in *in vitro *models devoid of BBB [49-51]. Therefore, the protective effects of caffeine against high cholesterol diet induced increases in astrocyte activation and increases in density of microglia might parallel its protective effects against BBB disruption.

In the present study, detailed molecular mechanisms whereby caffeine protects against BBB disruption were not explored. But, we did measure plasma concentrations of caffeine. Plasma caffeine concentrations were in the range of 5 to 6 μM in rabbits that ingested caffeine at the dose of 3 mg/day. The method used in our study to quantify plasma caffeine levels, however, excluded protein-bound caffeine. The protein-bound caffeine has been shown to be about 35% of total caffeine levels in plasma [52]. Therefore, the total plasma caffeine concentrations might have been about 10 μM in our rabbit model. At this concentration, the only known targets of caffeine are adenosine receptors [38]. Thus, the most likely pharmacological effects whereby caffeine might be exerting its protective effects against BBB disruption are blocking adenosine receptors.

## Conclusion

We conclude that chronic ingestion of caffeine protects against high cholesterol diet-induced disruption of the BBB. BBB is essential for brain homeostasis and, once disrupted, can contribute to a variety of neurological disorders. Therefore, our findings that caffeine, a safe and readily available drug, can stabilize BBB have important implications for therapeutic interventions against neurological disorders. Furthermore, it might be important to control ingestion of caffeine and drugs similar to caffeine in patients enrolled in clinical trials and/or prescribed therapeutic strategies where selective opening of the BBB to facilitate drug entry into brain is desired. Further detailed studies are now warranted to determine detailed mechanism(s) by which caffeine protects against BBB disruption, whether these effects on BBB are species-specific, and whether pathophysiological factors in addition to high dietary cholesterol that are related to acute and chronic neurodegenerative diseases are implicated.

## Competing interests

The author(s) declare that they have no competing interests.

## Authors' contributions

XC participated in the design of the study, carried out immunoblotting, immunostaining studies and Evan's blue leakage assays, participated in data analysis, and drafted the manuscript. JWG carried out caffeine level measurements, participated in data analysis, and helped draft the manuscript. JFW carried out caffeine level measurements, participated in data analysis. OG carried out cholesterol level measurements, participated in animal care and sample preparation, contributed reagents, participated in data analysis, and helped draft the manuscript. JDG conceived of the study, participated in the design of the study, participated in data analysis, and helped write the manuscript. All authors read and approved the final manuscript.
