# Correction: Caffeine blocks disruption of blood brain barrier in a rabbit model of Alzheimer's disease

**DOI:** 10.1186/s12974-023-02725-w

**Published:** 2023-02-18

**Authors:** Xuesong Chen, Jeremy W. Gawryluk, John F. Wagener, Othman Ghribi, Jonathan D. Geiger

**Affiliations:** Department of Biomedical Sciences, School of Medicine and Health Sciences, University of North Dakota, 504 Hamline Street, Grand Forks, ND 58203 USA

**Correction** **:** **Journal of Neuroinflammation (2008) 5:12** **https://doi.org/10.1186/1742-2094-5-12**

Following publication of the original article [1], the authors identified an error in Fig. 4. The correct figure is given in this correction (Fig. 4).

**Fig. 4:**
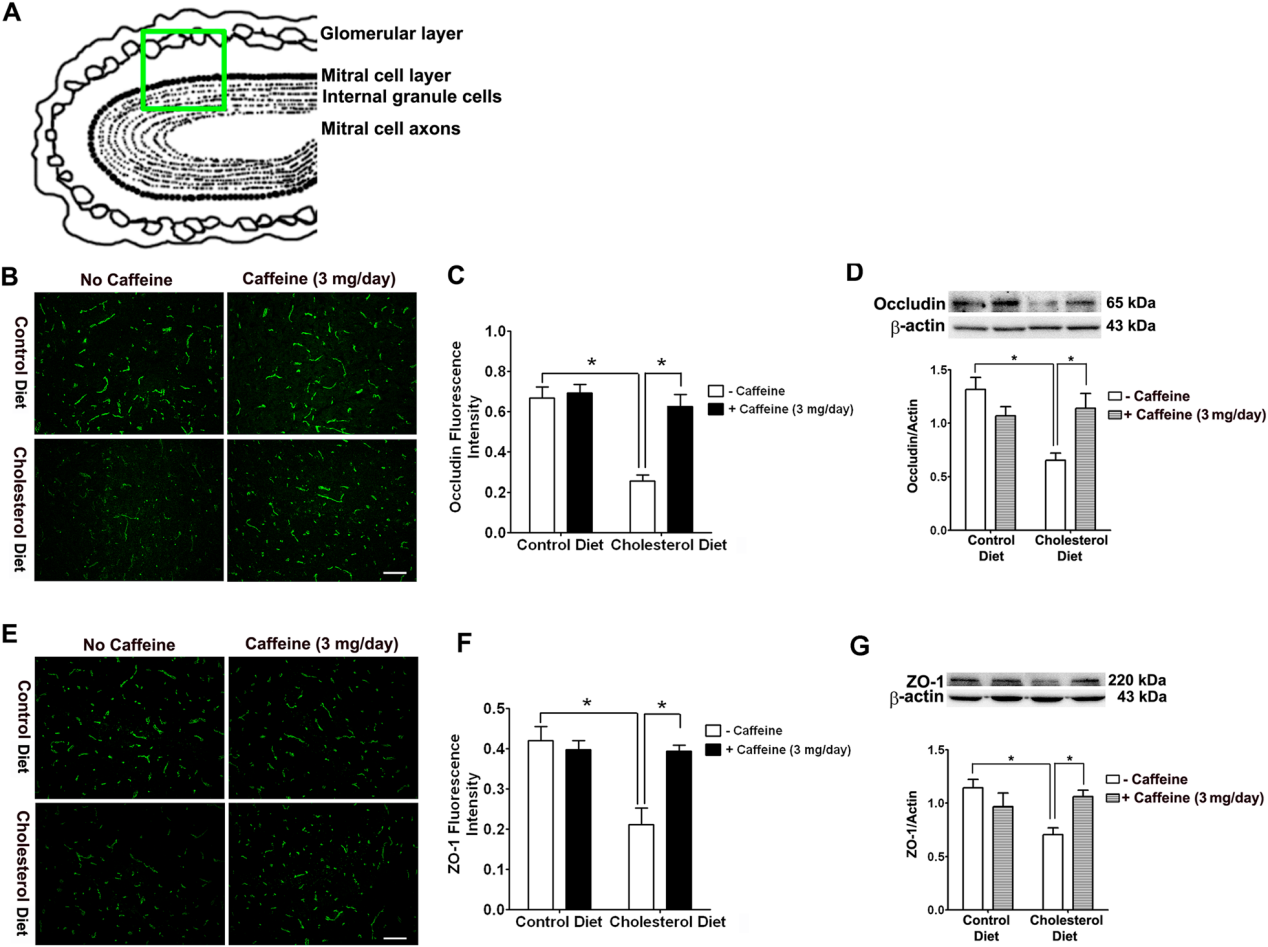
Caffeine blocks high cholesterol diet-induced down-regulation of tight junction proteins Caffeine blocks high cholesterol diet-induced down-regulation of tight junction proteins. **A** Schematic draft of a section of olfactory bulb, the green square indicated where the fluorescent images were taken for measures of the expression of occludin and ZO-1. **B** Decreased occludin immunostaining was observed in olfactory bulb from cholesterol-fed rabbits and this effect was blocked by caffeine. Caffeine alone had no effect on occludin immunostaining in normal rabbit brain. Representative images taken from 2 rabbits in each group with 6 sections from each animal are shown. Bar = 100 µm. **C** Quantitative data from B shows that high cholesterol diet significantly decreased occludin immunopositive staining in olfactory bulb, and this effect is blocked by caffeine. **D** Cholesterol-enriched diet decreased significantly protein levels of occludin, and these effects were blocked by caffeine at the dose of 3 mg/day. Caffeine alone did not significantly change protein levels of occludin in normal rabbit olfactory bulb (n = 4, *p < 0.05). **E** Decreased ZO-1 immunostaining was observed in olfactory bulb from cholesterol-fed rabbits and this effect was blocked by caffeine. Caffeine alone had no effect on ZO-1 immunostaining in normal rabbit brain. Representative images taken from 2 rabbits in each group with 6 sections from each animal are shown. Bar = 100 µm. **F** Quantitative data from **E** shows that high cholesterol diet significantly decreased ZO-1 immunopositive staining in olfactory bulb, and this effect is blocked by caffeine. **G** Cholesterol-enriched diet decreased significantly protein levels of ZO-1, and these effects were blocked by caffeine at the dose of 3 mg/day. Caffeine alone did not significantly change protein levels of ZO1 in normal rabbit olfactory bulb. n = 4, *p < 0.05

In Fig. 4E of the original article [1], the same image was mistakenly copied twice into the final figure of fluorescent images of the tight junctional protein ZO-1 in cholesterol-fed and control rabbits treated with caffeine. The fluorescent image from control diet plus caffeine (3 mg/day) group was inadvertently copied twice into the final figure. In this correction, the correct representative fluorescent image from cholesterol diet plus caffeine (3 mg/day) group was inserted into Fig. 4E.
